# Effect of locomotor demands on cognitive processing

**DOI:** 10.1038/s41598-019-45396-5

**Published:** 2019-06-25

**Authors:** J. Cortney Bradford, Jamie R. Lukos, Antony Passaro, Anthony Ries, Daniel P. Ferris

**Affiliations:** 1 Human Research and Engineering Directorate, U.S. Army Research Laboratory, Aberdeen Proving Ground, Aberdeen, MD United States; 2 Cyber, Science & Technology Department, Naval Information Warfare Center Pacific, San Diego, CA United States; 3 J. Crayton Pruitt Family Department of Biomedical Engineering, University of Florida, Gainesville, FL United States; 4 Warfighter Effectiveness Research Center, Dept. of Behavioral Sciences and Leadership, United States Air Force Academy, CO, United States

**Keywords:** Electroencephalography - EEG, Decision

## Abstract

Understanding how brain dynamics change with dual cognitive and motor tasks can improve our knowledge of human neurophysiology. The primary goals of this study were to: (1) assess the feasibility of extracting electrocortical signals from scalp EEG while performing sustained, physically demanding dual-task walking and (2) test hypotheses about how the P300 event-related potential is affected by walking physical exertion. Participants walked on a treadmill for an hour either carrying an empty rucksack or one filled with 40% of their body weight. During the walking conditions and during a seated control condition, subjects periodically performed a visual oddball task. We recorded scalp EEG and examined electrocortical dynamics time-locked to the target stimulus. Channel-level event-related potential analysis demonstrated that it is feasible to extract reliable signals during long duration loaded walking. P300 amplitude was reduced during loaded walking versus seated, but there was no effect of time on task. Source level activity and frequency analysis revealed that sensorimotor, parietal, and cingulate brain areas all contributed to the reduced P300 amplitude during dual-task walking. We interpret the results as supporting a prioritization of cortical resources for walking, leading to fewer resources being directed toward the oddball task during dual-task locomotion.

## Introduction

Humans often experience situations that require execution of multiple tasks at once. Dual-tasking locomotor and cognitive tasks can result in cognitive-motor interference, where participants adopt altered strategies for performing the dual tasks^1^ that can result in suboptimal task performance^2,3^. Despite these known behavioral changes, the underlying neural mechanisms associated with this interference remain unclear. Understanding the neural basis of cognitive-motor interference may have implications for mitigating or predicting decrements in task performance. In addition, having a better understanding of how dual-tasking can influence underlying cognitive dynamics may also improve technologies for brain-computer interfaces (BCIs). Developing a brain-computer interface that can still operate when the user is multi-tasking would add value to the technology for real world use.

Recent advancements in non-invasive electroencephalography (EEG) hardware and signal processing techniques have allowed for better extraction of neural signals during locomotor activities^4–6^. This has enabled researchers to investigate the neural mechanisms underlying cognitive-motor dual tasking during walking. Previous studies have looked at electrocortical dynamics during dual-tasking walking over a range of speeds^7^, and even dual-task running^5^. Though cortical changes are likely dependent on the cognitive task performed, some studies have found changes in neural dynamics when cognitive tasks are performed during walking compared to seated conditions^8–10^. These results may indicate adaptive neural processing strategies that optimize performance under dual-task conditions. However, there are many real-world locomotion variants that have not been explored in dual-tasking situations.

In dismounted military scenarios, it is not uncommon for individuals to walk for long durations with rucksack loads. Load carriage is a critical task for deployed military personnel but has been shown to impact cognitive performance during locomotion^11–13^. This work has greatly added to our understanding of behavioral outcomes and subjective assessments, but there has been limited research on neural mechanisms. Level of physical exertion and duration of cycling has been shown to affect cognitive neural dynamics^14–17^, however it remains unclear if this will translate to a dual-task walking paradigm.

One theory is that the cognitive and motor tasks compete for cortical resources and that the motor task draws resources away from the cognitive task^18^. DeSanctis *et al*. demonstrated that walking altered cognitive task ERP amplitude and topography relative to a seated condition. Additionally, several studies have demonstrated that increased physical exertion requires additional cortical activation^14–16^. Thus, as the locomotor task becomes more demanding, such as increasing physical demand with a load, more cortical resources may be allocated to the locomotor task. There is also some evidence that physical fatigue during a locomotor task may recruit more cortical resources as well^19^. The P300 ERP component is well suited to testing this theory, as the amplitude is thought to index the amount of resources that are allocated to stimulus processing^10,20,21^. Additionally, a source-based analysis could help uncover which cortical areas are involved and how their activity changes in response to manipulation of the locomotor task^22,23^.

We also expect that adding a heavy rucksack will alter the signal to noise ratio of EEG. There are many sources of artifacts (e.g. movement, muscle, sweat) that can contaminate EEG data and affect/limit the interpretation of underlying brain function^5,24,25^. While several studies have demonstrated the viability of using EEG to record brain function during motion, including locomotion, there are variants that induce a signal to noise level that limits interpretability^6,24,26,27^. It is well documented in the literature that heavy load carriage changes locomotor biomechanics, including ground reaction forces and muscle activity^28^. These altered biomechanics have the potential to affect the artifact recorded in EEG during sustained loaded walking and it is unknown how this will impact interpretability of the data.

The primary goals of this study were to: (1) assess the feasibility of extracting electrocortical signals from scalp EEG data while performing a sustained, physically demanding walking task, and 2) test hypotheses about the electrocortical dynamics for walking with and without rucksack load across time. Participants walked on a treadmill for an hour either carrying an EEG system on an empty rucksack or one filled with a load similar to that used by Soldiers in training (40% body weight). While walking, participants performed a decision-making task that required responses to target images displayed on a monitor in front of them (a modified version of the classic visual oddball paradigm). Additionally, participants performed the same cognitive task while seated before and after walking. We hypothesized that: (1) increased physical load would alter cortical processing and result in differences between loaded and unloaded conditions, and (2) over time, the addition of a demanding physical task would alter the cortical responses to the cognitive task, particularly in the cortical regions dedicated to sensorimotor function. To test these hypotheses, we implemented standard channel-level event-related potential (ERP) analyses and state-of-the-art source-level event-related spectral perturbation (ERSP) analyses. We included the standard channel level analysis so that we could compare our results to what has been well documented in the P300 literature, we included the newer source level analysis to better elucidate which cortical areas were affected by the dual tasking paradigm^4,20–22^. This work is an important step towards understanding cortical mechanism in more realistic settings as well as fielding physiological monitoring and BCI technologies in real world environments.

## Methods

### Participants

Twenty-five young, healthy participants took part in this study. Two participants did not complete the study. One participant withdrew after the first testing session, but did not give a reason for withdrawal. One participant reported knee pain and participation was terminated by the research staff. The data from five other participants were corrupt or incomplete due to hardware/software issues. Thus, we obtained useable data from eighteen participants (11 males, 7 females; age 26.5 ± 6 years). Participants were screened prior to participation to ensure the absence of musculoskeletal, neurological, and cardiovascular conditions that would limit their ability to safely complete testing and were deemed in good physical shape such that they could walk on a treadmill for an hour with 40% of their body weight. Although we did ask questions during screening about how often they trained with a heavy backpack, we did not exclude participants based on their experience carrying heavy loads.

This study was reviewed and approved by the Institutional Review Board of the U.S. Army Research laboratory. The procedures were carried out in accordance to the applicable human subjects U.S. federal regulations. Prior to testing, all participants signed an informed consent document approved by the human subject Institutional Review Board of the U.S. Army Research Laboratory.

### Tasks and stimuli

#### Physical task

Participants walked at 1.0 m/s (0° incline) on an instrumented treadmill (Model DBCEEWI-0509, AMTI, Watertown, MA) for 60 minutes under two physical conditions. In the low physical demand condition participants carried an empty Modular Lightweight Load-carrying Equipment (MOLLE) II rucksack with only the components associated with the EEG data collection (battery and A/D box) added to the rucksack (~13 lbs. combined). In the high physical demand condition, we added lead pellets to the rucksack to bring the total load up to ~40% body weight. The 40% body weight load was chosen to be within the range of real world loads that Soldiers are trained to carry and often may carry in real operations^12^ (U.S. Army Field Manual 21–18).

#### Cognitive task

The cognitive task was a modified version of a classic, two-stimulus visual oddball paradigm^29^. The neural responses produced by the oddball paradigm are robust and well-characterized^20,30^ and thus provided a good baseline for establishing that EEG activity can be meaningfully resolved in a high-artifact scenario. The target and non-target stimuli were presented on a computer screen positioned about 24 inches in front of and centered at the participant’s eye level. Each stimulus appeared in the center of the screen for 150 ms followed by a jittered 1500–2000 ms inter-stimulus interval during which only a fixation cross was displayed. The target (12% frequency) was a computerized depiction of an enemy insurgent holding a weapon and the non-target (88% frequency) was a computerized depiction of a Soldier also holding a weapon. We instructed participants to press their hand-held button as fast as they could when they saw the rarely-occurring target. This task was structured in 6, ~3 min intervals interspersed with self-paced breaks, resulting in a total of ~20 min per instance of task administration. Participants completed the task 4 times, including once before walking in a seated posture, twice during walking, and once after walking also in a seated posture. We chose a seated control condition for the cognitive task since it is in line with classic EEG laboratory testing and is the posture most often cited in the literature. We considered including a bout of the cognitive task with the participant in a standing posture, but ultimately did not want to require participants to stand for the required 20 mins after walking for an hour with a heavy load or introduce potential confounds due to balance requirements during standing.

### Protocol

Participants visited the lab for three sessions: an initial training/screening session and two data collection sessions. All sessions occurred on different days within a 14-day period and with at least one rest day between them. During the initial training/screening session, participants practiced all study tasks until they felt comfortable. Participants returned to the testing facility two additional times to perform the Loaded and Unloaded physical conditions on separate days, with the order of days counterbalanced across participants. During those sessions, a 256 channel EEG system (Biosemi, Amsterdam, Netherlands) was applied to the participant’s head with 8 external electrodes applied to the neck. Each EEG electrode position was digitized using the Zebris ELPOS system (Zebris Medical GmbH, Denmark) to improve source localization accuracy.

#### Data collection

Testing sessions consisted of 3 parts (Seated Pre-walking, Walking, and Seated Post-walking) lasting approximately 2 hours in total. During the seated pre-walking phase, participants sat in a chair with the monitor adjusted to about eye level. Participants performed the visual oddball task while seated. Participants then stood up, we adjusted the monitor to eye level, and experimenters helped participants put on the rucksack. Participants then walked on the treadmill for 1 hour. Participants performed the cognitive task twice during walking (starting at minute 4 and at minute 35 of walking, approximately 20 minutes in length). The remainder of the time participants fixated on the crosshairs in the middle of the screen. EEG data were collected continuously during the entire testing session. Additionally, we used the Borg “Rating of Perceived Exertion” to measure participants’ perceived physical activity intensity level (Borg, 1982). It is a scale that ranges from 6 (no exertion at all) to 20 (Maximal exertion). We recorded rating of perceived exertion before and after each instance of the cognitive task by showing the participant the scale and having them verbally report the number that most closely resembled how they felt at that time.

### Data processing

#### Behavioral and psychological data processing

We computed reaction time as the time between the target stimulus presentation and the moment at which participants pushed the button with their right thumb. Reaction times were only computed for correctly identified target trials. We computed accuracy as the percentage of correctly identified targets. Finally, we computed mean rating of perceived exertion scores for each condition by averaging all of the scores recorded for each condition. Statistical differences between conditions for each measure were computed using a one-way, repeated-measures ANOVA.

#### EEG data preprocessing

**Figure 1:**
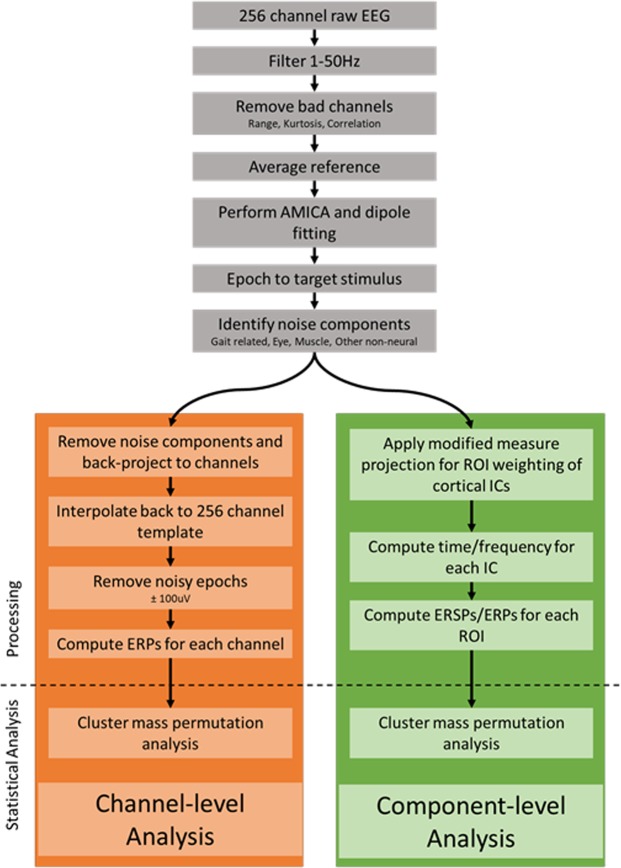
Data processing pipeline. Data processing workflow. EEG data were first processed using fairly standard cleaning methods (grey boxes). After running AMICA, the data were processed in two different ways in order to perform both the channel-level analysis (orange boxes) and component-level analysis (green boxes).

We analyzed data offline using custom Matlab (Mathworks, Natick, MA) scripts and EEGLAB^31^. An outline of the processing pipeline is depicted in Fig. 1. First, we bandpass filtered the data between 1–50 Hz then we removed bad channels using standard statistical thresholds (i.e., range, probability, standard deviation, kurtosis). Next, we computed the average reference using the remaining channels and applied an adaptive mixture independent component analysis (AMICA)^32,33^ to the cleaned channel time series to parse the data into spatially fixed, maximally temporally independent component signals^23^. The DIPFIT^34^ function in EEGLAB modeled each independent component as an equivalent current dipole within a boundary element head model based on the Montreal Neurological Institute standard brain (Quebec, Canada). We identified components as noise if their best-fit equivalent current dipole accounted for less than 80% of the variance seen at the scalp or if their scalp map, spectra, or activity were indicative of gait artifact, eye artifact, muscle artifact, or other non-neural activity^24,35–38^. To generate event-related potentials, we removed bad components as identified above and back projected the remaining components to the channels. Next, we interpolated the channels that were previously removed using the remaining cleaned channels to produce a consistent 256 channel montage for each subject. We epoched the cleaned data time-locking to stimulus presentation (−1 to 2 s) and performed a baseline-correction to a −200–0 ms pre-stimulus period. Subsequently, we removed noisy epochs identified using a threshold of ±200 µV. Finally, target and non-target ERPs were computed by averaging across remaining epochs at each channel for each subject.

#### Channel ERP statistical analysis

We determined the location and timing of significant differences between ERPs of each condition by submitting pair-wise data to a repeated measures, two-tailed cluster mass permutation t-test^39^ using a family-wise alpha level of 0.05 implemented in Fieldtrip^40^. All time points (512 in total) from 0–1000 ms at all 256 scalp electrodes were included in the test resulting in 131,072 total comparisons. 1000 permutations were used to estimate the distribution of the null hypothesis as it is the number recommend by Manly^41^ for a family-wise alpha level of 0.05. This permutation test analysis was used in lieu of more conventional analysis because it allowed us to test for differences in amplitude, topography, and timing of the event related potential while correcting for the large number of multiple comparisons. We chose the cluster mass statistic because it has been suggested to have good power for event related potentials such as the P300^42,43^. To determine feasibility of detecting expected differences in brain responses during the physically demanding task, we tested for differences between the non-target and target ERPs during the Loaded condition. To determine significant differences in neural dynamics related to target identification between the three conditions (Seated, Unloaded, Loaded) we tested for pair-wise differences in the target ERPs with cluster α = 0.017, adjusted for multiple comparisons.

#### Source localization ERP and ERSP analyses

Only non-artifact independent components with a residual variance below 20%, and with an equivalent dipole location within the Montreal Neurological Institute brain model volume were included in this analysis. We epoched all independent components around the target stimuli (−1 to 2 s) and then computed spectrograms. We estimated oscillatory power using a multitaper, Fast Fourier time–frequency transformation^44^ with frequency-dependent Hanning window tapers (time window: 1/log(f)). We calculated power for 2–40 Hz in steps of 0.25 Hz. We linearly mixed the independent components based on dipole source location using a modified measure-projection analysis^45,46^ to isolate activity in 56 regions of interest as specified by the Loni atlas^47^. We used the modified measure-projection approach to represent the equivalent dipoles for each independent component as a three-dimensional Gaussian density function. We set the standard deviation of the Gaussian distribution to 12 mm. We computed the independent component contribution to a given region of interest by determining the overlap between the region of interest and the dipole density function for that independent component and normalized such that each dipole could only be represented at unity across all 56 regions. We then computed the event-related spectral perturbation (ERSP) and event related potential (ERP) for each region of interest by summing the relative contribution of each independent component to a given region of interest. This was performed for each subject separately, such that each subject had an ERSP and ERP for each region, if activity was found in that region. We determined timing, location, and frequency of significant differences between ERSPs by submitting pair-wise comparisons to a repeated measures, two-tailed cluster mass permutation t-test with the family-wise alpha set to 0.05 with the factors region of interest (56 regions), time (0–1 s, 115 bins), and frequency power (2–40 Hz, 151 bins) for a total of 972,440 comparisons. Similarly, we determined differences in source ERPs between conditions by submitting data to a repeated measures, two-tailed cluster mass permutation test (t-test) with the family-wise alpha set to 0.05 with the factors region of interest (56), time (0–1 s, 512 time points) for a total of 28,672 comparisons. We set the number of permutations to 1000 and cluster α = 0.017 to account for multiple comparisons for both ERSPs and ERPs. Note that *n* = 17 rather than 18 for this test due to one outlier whose ERPs were many magnitudes larger than all other participants.

## Results

### Psychological and behavioral results

**Figure 2:**
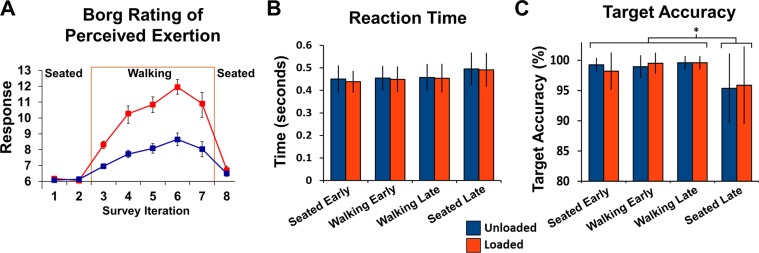
Psychological and Behavioral Results. Psychological and Behavioral Results. Panel A shows subjects responses to the BORG Rating of Perceived Exertion over time. It was administered before and after every bout of the cognitive task. Panel B shows the reaction time to respond to the visual target during the seated (early and late) and walking (early and late) cognitive task bouts. Panel C depicts the Target Accuracy for the same cognitive bouts as Panel B.

As expected, rating of perceived exertion significantly increased with increasing physical load (Fig. 2A). The seated condition had a mean score of 6.2 ± 0.4, which falls in the range of “no exertion at all” to “extremely light”. The unloaded walking condition had a mean score of 7.9 ± 1.2 which falls in the range of “extremely light” to “very light”. The loaded walking condition had a mean score of 10.3 ± 1.8 which falls in the range of “very light” to “light”. There was a significant main effect of condition on the Borg rating of perceived exertion (F(2,42) = 89.086, p < 0.001,η_p_^2^ = 0.809). Post-hoc pairwise comparisons using a Bonferroni corrected alpha <0.017 revealed significant differences between each combination of conditions (Table 1). Unloaded walking had a significantly greater rating of perceived exertion than seated (p < 0.001). Loaded walking had the largest rating of perceived exertion, and was significantly greater than both seated and unloaded walking (p < 0.001 for both comparisons,). There was a significant main effect of condition on reaction time (Table 1; F(2, 34) = 3.658; p = 0.036, η_p_^2^ = 0.177), however pairwise comparisons were not significant. There was a significant main effect of condition on accuracy (Table 1; F(2, 34) = 10.484; p < 0.001, η_p_^2^ = 0.381). Accuracy was significantly lower during seated compared to unloaded and loaded walking (p = 0.015 and p = 0.006 respectively) but not significantly different between loaded and unloaded walking (p = 0.140).

**Table 1:** Behavioral results.

	Seated	Unloaded	Loaded
	Mean ± SD	Mean ± SD	Mean ± SD
Rate of Perceived Exertion	6.23 ± 0.35^U,L^	7.85 ± 1.17^S,L^	10.38 ± 1.75^S,U^
Reaction Time (ms)	468.8 ± 59.97	455.9 ± 55.55	451.4 ± 59.38
Accuracy (%)	97.17 ± 3.31^U,L^	99.26 ± 1.30^S^	99.55 ± 1.13^S^

^S^Significant difference from Seated.

^U^Significant difference from Unloaded.

^L^Significant difference from Loaded.

### Feasibility assessment

**Figure 3:**
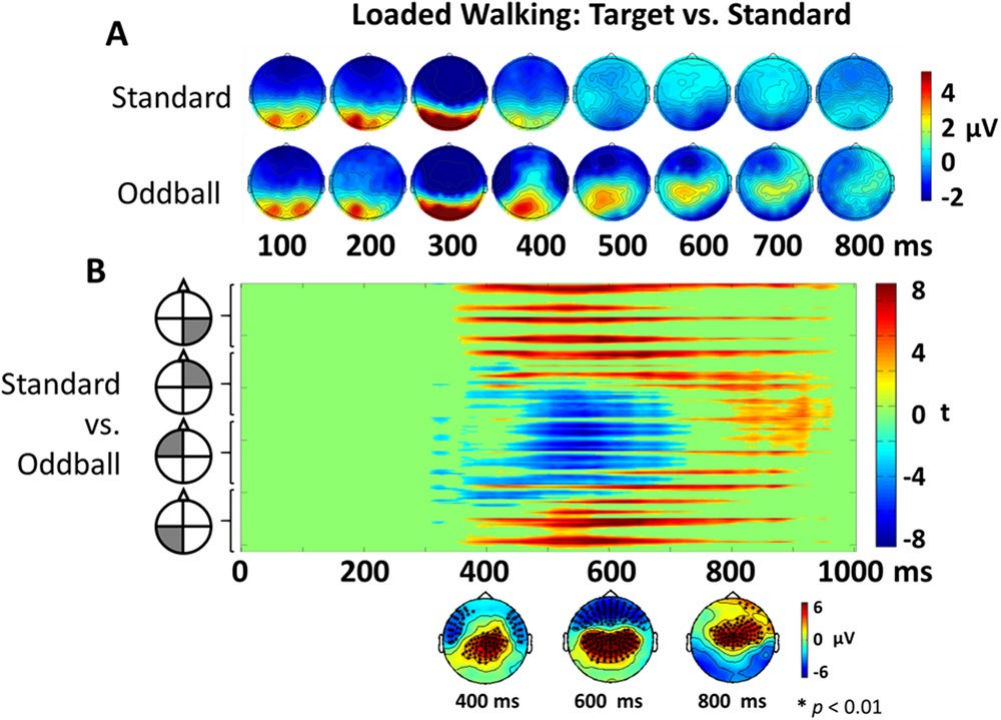
Feasibility of measuring ERPs during loaded walking. Panel A shows topographical plots of the channel-level ERPs across time for non-target vs. target of the loaded walking condition. Data were time-locked to the presentation of the stimulus. Panel B displays the t-scores testing the significant differences between the non-target and target trials across all channels using cluster mass permutation. Channels are ordered based on the 256 Biosemi electrode cap (A1 – H32) and displayed by quadrant. Non-significant values were set to zero (green). The topographical plots below the figure represent the spatial location of the ERP t-test results for 400, 600 and 800 ms. The black symbols denote channel location and levels of significance (p < 0.01) for significantly different ERP responses.

We found significant differences between non-target and target ERPs during the Loaded walking condition (Fig. 3). The potentials had typical early (100 ms) increases in amplitude over the occipital region, but diverge between 300 and 400 ms post-stimulus. At 400 ms, a clear increase in amplitude over the centro-parietal area occurred for the target stimuli but not for the standard. There was also a decrease in amplitude over the frontal regions for the target stimulus but not the non-target. We submitted the data to a cluster mass permutation test and found significant differences between the target and non-target stimuli as demonstrated by Fig. 3B. Around 400 ms post stimulus, there was a significant cluster near the sensorimotor and parietal regions. Between 400 and 800 ms after stimulus presentation, the cluster moved slightly anterior (Fig. 3B topoplots on bottom). We also found a significant negative cluster over the frontal region between about 350 ms and 700 ms.

### Channel-level event related potentials

**Figure 4:**
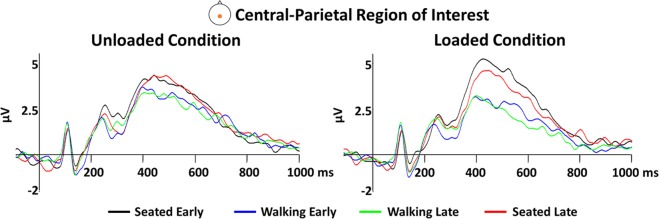
Early and late task channel-level ERPs. Channel-level ERPs over time. The plots show ERP traces averaged across subjects in the central-parietal region of interest for the seated (early and late) and walking (early and late) cognitive bouts for the unloaded and loaded conditions (left and right panels, respectively).

We did not find any statistical differences in the amplitude of early and late event-related potentials in either the seated or walking conditions (Fig. 4), suggesting that time on task did not have an effect on cognitive processing. Given this, all following channel level and cortical source analyses are collapsed into conditions called Seated, Loaded and Unloaded.

**Figure 5:**
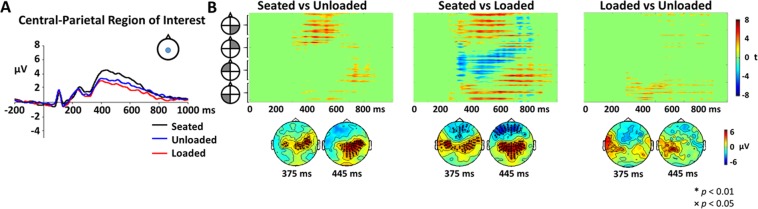
Channel-level ERPs across conditions. Panel A displays the channel-level ERP in response to the target stimulus averaged across all subjects during seated, unloaded, and loaded conditions for the Central-Parietal ROI (see location shown in panel inset). Panel B shows the pair-wise differences between all three levels of locomotor demand (Seated, Unloaded, Loaded), α = 0.017 to account for multiple comparisons. Non-significant cluster t-values were set to zero (green). The topographical plots below each figure show the spatial location of the ERP responses for 375 and 445 ms. The black symbols denote channel location and levels of significance **p < 0.01;×p < 0.05) for significantly different ERP responses.

Analysis of the event-related potentials at the channel level revealed large differences between walking and seated conditions, and some small but significant differences between unloaded and loaded conditions. Figure 5A shows grand average ERPs for each condition at a central-parietal region comprised of 12 channels surrounding 10–20 electrode location Pz. Unloaded and Loaded walking condition ERPs demonstrate early perceptual components very similar to the Seated condition. Around 350 ms all three curves demonstrate a positive deflection as expected for a P300 response to the target, however both the unloaded and loaded walking conditions appear to have a lower amplitude than the seated condition between ~350–700 ms. The loaded walking condition appears to have an even smaller amplitude than the unloaded walking condition in the same time frame. By applying a cluster mass permutation test, we were able to determine significant differences between the amplitude of each condition across the entire scalp (all channels) over the duration of the ERP (0–1000 ms). Significance-masked t-values are shown across time for all channels in the top plots of Fig. 5B and significant channels are displayed on topological plots in the bottom plots for relevant time points. We found that there were significant differences, particularly in scalp areas near the sensorimotor and parietal regions. Temporally, the differences occurred during the cognitive processing and response stages of the ERP (~300–800 ms) with no differences present during perceptual processing (0–300 ms) across all conditions. Spatially there were significant differences across conditions. Specifically, differences (p < 0.01) between the seated and unloaded conditions were focused on the sensorimotor and parietal regions (most prominently around 375 and 445 ms, respectively), with a bias towards the right side of the head (Fig. 5B, left plot). The amplitude of the responses to the oddball target were smaller in the unloaded compared to the loaded conditions in these areas. We found similar differences when comparing seated and loaded in the sensorimotor and parietal regions, but with differences also present on the left side of the brain, thus less lateralization was found when comparing the seated and loaded conditions. Unlike in the sensorimotor and parietal regions, the amplitude of the response in the frontal regions was greater during the loaded walking compared to seated condition. When we tested for differences between the loaded and unloaded conditions, we found small sparse differences in electrodes near the left sensorimotor area from ~300–800 ms (Fig. 5B, right plot). Overall, the channel level results suggest changes in cortical dynamics of the brain during target detection under varying levels of locomotor demand.

### Component-level event related potentials

**Figure 6:**
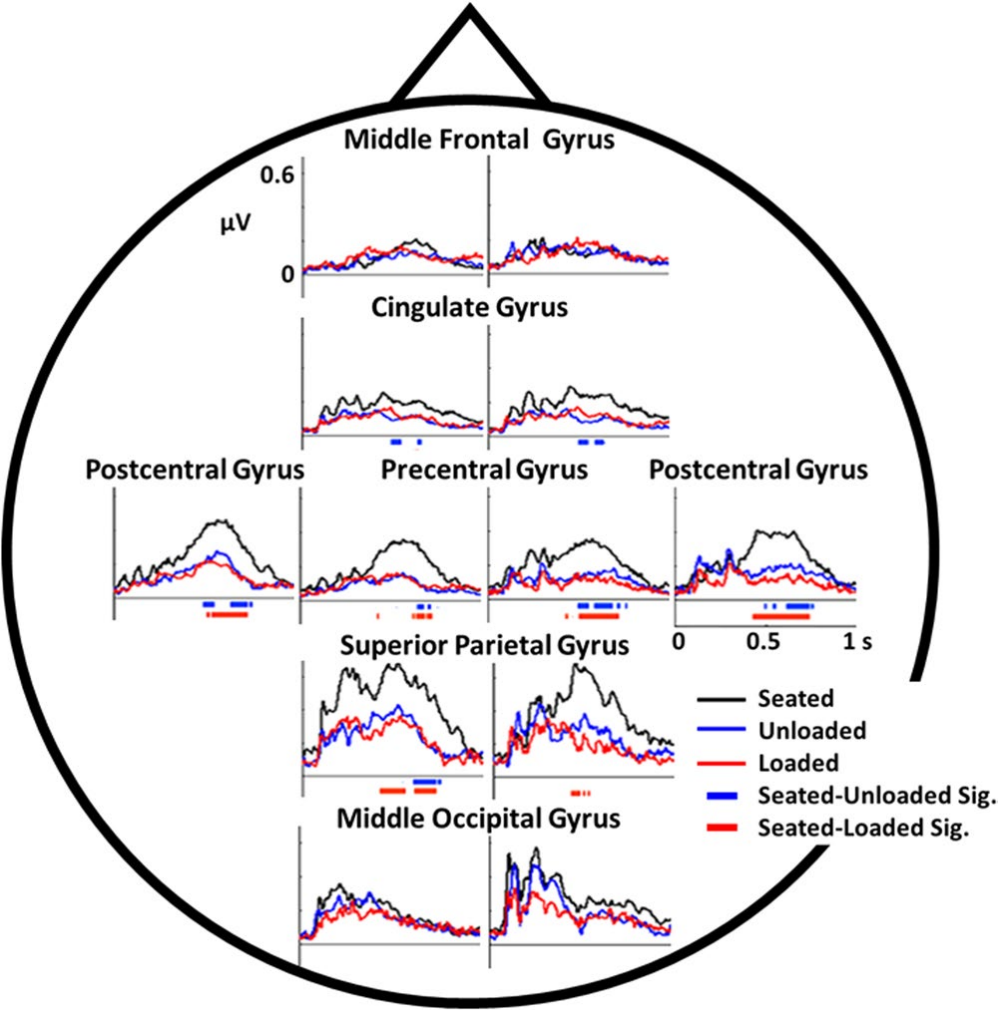
Component-level ERPs across conditions. Each plot depicts the average component-level ERPs for the Seated, Unloaded, and Loaded conditions in the approximate location of 12 ROIs across the scalp. Under each plot, the red and blue bars denote significant differences between Seated vs. Unloaded (blue bar) and Seated vs. Loaded (red bar) over time as determined by the cluster mass permutation test. No significant differences were found between the Unloaded and Loaded conditions.

We found significant differences in the sensorimotor (precentral/postcentral gyri), parietal (superior parietal gyrus), and cingulate (cingulate gyri) regions when comparing the seated to the unloaded and loaded conditions (Fig. 6; red and blue bars under each graph, respectively) ~300–800 ms after stimulus presentation. Differences between seated and walking conditions were more robust in the left with respect to right superior temporal gyrus (Fig. 6; solid red and blue bars vs sparse red and blue bars).

The ERPs in the middle occipital gyrus region of interest were similar across all three conditions and had the greatest amplitude in the early perceptual period (0–300 ms) with little activity during the cognitive period (>300 ms). Superior parietal gyri demonstrated activity throughout the ERP with the seated waveform demonstrating the greatest amplitude during the cognitive period (>300 ms). The seated condition ERP had nearly twice the amplitude during this time period compared to both the unloaded and loaded walking conditions. Precentral and postcentral gyri ERPs were low amplitude in the early period (<300 ms) with a later increase in amplitude around 400 ms. Both the unloaded and loaded conditions have smaller amplitudes than the seated condition during the later cognitive period (>300 ms) and not the perceptual period (<300 ms). There was little stimulus-related activity in the cingulate gyrus, however the unloaded condition waveform had decreased amplitude compared to the seated condition during the later cognitive period (>300 ms). The middle frontal gyrus ERPs had the lowest stimulus-related amplitude of all the regions of interest and there were no differences between waveforms for each condition. We did not detect any significant differences between the loaded and unloaded condition ERPs for any regions of interest.

### Component-level spectral fluctuations

**Figure 7:**
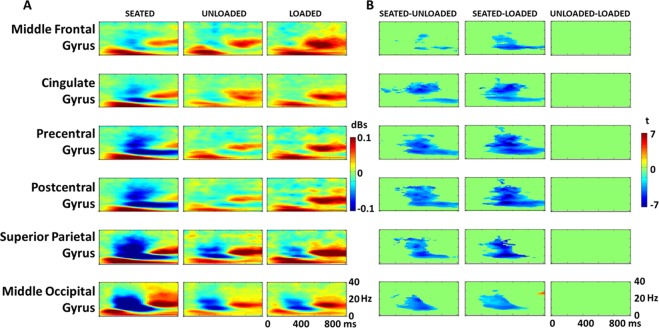
Component-level ERSPs. Panel A displays the event related spectral perturbations (ERSPs) for the seated, unloaded, and loaded conditions (left, right and middle columns, respectively) for six relevant ROIs across the scalp. Panel B shows the significance-masked cluster mass permutation results for Seated compared to Unloaded and Seated compared to Loaded conditions (left and right columns, respectively) for the same ROIs as in Panel A. Non-significant cluster t-values were set to zero (green). No significant differences were found between the Unloaded and Loaded conditions. Note that these ROIs are the same as those depicted in Fig. 4, but only data from the right side of the brain is shown here due to the lateral similarities found across the scalp.

There was more cortical desynchrony during the seated condition compared to the walking conditions (Fig. 7), particularly in the precentral, postcentral, parietal and occipital regions and in the alpha (8–13 Hz) and beta (13–30 Hz) bands. Since the left and right side ERSPs were nearly mirror images of each other, we have presented only the six regions of interest from the right side of the cortex here. This desynchrony starts at approximately 200 ms in the occipital and parietal regions and approximately 300 ms in the postcentral, precentral, and cingulate areas. During both the unloaded and loaded walking conditions (Fig. 7A, middle and right columns), the alpha and beta desynchrony that was present in the precentral and postcentral regions in the seated conditions disappears. Alpha and beta desynchrony is still present in the parietal and occipital areas, but to a much lesser degree than in the seated condition.

Also noteworthy is the alpha synchrony (Fig. 7A, red areas in 8–13 Hz range) occurring later in the trial at approximately 500–600 ms. This activity is more prominent in the walking conditions (particularly the loaded condition) in the frontal, cingulate, precentral, and postcentral. Late beta synchrony in the parietal and occipital areas is common to all three conditions. In all conditions, there was a strong theta band (4–7 Hz) synchrony that starts very early after the trial onset (<100 ms).

Statistically, we found significantly stronger alpha and beta band desynchrony during the seated condition compared to both walking conditions generally starting at about 300 ms (Fig. 7B). The differences were overall more prominent (larger clusters, longer periods of time) in the cingulate gyrus, pre and postcentral gyri, and the superior parietal gyrus compared to the more frontal and occipital regions. The difference in the alpha band extended to the end of the trial for the frontal, cingulate, precentral and postcentral areas. Significant differences in the beta band tended to occur between 300 and 600 ms. There were no significant difference between the loaded and unloaded condition (Fig. 7B, right plots). However, there were larger differences between the loaded and seated condition than between the unloaded and seated condition (darker blue in Fig. 7B middle plots vs. left plots).

## Discussion

The ability to measure electrocortical dynamics related to a simple visual cognitive task was no more difficult during walking with a heavy rucksack load than it was during walking without a rucksack load. We detected a reliable P300 event-related potential during both walking conditions and during the seated condition. While there was no change in cognitive task performance across conditions, there were changes in brain dynamics required to execute dual-tasking. Our data suggest that multiple brain regions contribute to the reduction in P300 amplitude during cognitive-motor dual-tasking. The brain areas that demonstrated reduction in activity during walking compared to seating were focused in the parietal, cingulate, precentral, and postcentral gyri. By comparison, the changes in the frontal and occipital regions between seated and walking conditions were minimal. Importantly, it was the source level analysis with independent component analysis rather than the electrode channel analysis that provided the insight about the relative contributions of different brain areas to the reduction in the event-related potential.

### Feasibility

Our paradigm simulated an operationally-relevant vigilance task (i.e., target detection and discrimination) that required sustained mental effort, a characteristic essential for Soldiers in the field while walking with heavy loads. Recording EEG during this type of physically demanding task has not previously been performed and is often avoided due to the possibility of motion artifacts and muscle activity corrupting the electrocortical signals^26,48,49^. While recording EEG during human movement has become more popular and has been shown to be useful for understanding brain function^4,5,7,9,27,50^, it cannot be assumed that EEG will record interpretable information in all movement scenarios. In studies where participants were walking while EEG was recorded, gait speed was often limited to slow speeds to constrain head movement. As movement parameters change (e.g., gait type, speed, terrain), EEG data can have different amounts of noise (i.e. muscle activity, motion artifact)^5,24,50^. Behavioral studies of walking with a heavily loaded rucksack have demonstrated changes in biomechanics of gait including ground reaction force, step length, body segment acceleration, and muscle activation^12,28,51,52^, all of which have been identified as sources of noise in EEG. Our results show that we were able to measure the expected waveforms and topography related to a visual discrimination task during walking with a heavily loaded rucksack. The data included a clear P300 event-related potential for target stimuli relative to non-target stimuli during loaded walking, which has become a benchmark for EEG data quality^4,53–55^. This supports our first hypothesis that it is feasible to detect a P300 during heavily loaded rucksack marching.

### Behavioral/psychological

The rating of perceived exertion supported an increased locomotion workload with the experimental manipulation of adding 40% bodyweight to the rucksack. The rating of perceived exertion was significantly different across the three conditions: seated, unloaded walking, and loaded walking. We did not include measures of biomechanics or energetics in our study, but there is ample evidence from past studies that the addition of 40% body weight load during walking substantially affects gait biomechanics and metabolic measures^12,28,51,52^. Although we did not measure it, we also observed increased amounts of sweating with the greater rucksack load. After one hour of walking with the loaded rucksack, subjects were often soaked with sweat under the rucksack and electrodes taped to the neck to record muscle activity were often falling off. This also included mastoid electrodes, requiring us to use an average reference for EEG data analysis.

We found minimal differences in cognitive task performance across conditions. There were no significant differences in reaction times across conditions and although there was a significant difference in accuracy, it was only a ~2% difference. Some studies on young healthy adults have demonstrated decreases in cognitive task performance during walking compared to not walking (e.g., seated or standing)^2,56–59^, but in most cases the differences are not large. There are other studies that have not found any differences in cognitive performance during walking compared to not walking^7,8,60^. In the scope of previous tasks used, the visual oddball task is not very demanding cognitively. We chose it primarily due to its clear and consistent event-related potential for study^4,54,55^. Previous loaded walking studies that have found reduced cognitive performance under certain circumstances. Eddy and colleagues^12^ studied subjects performing auditory and visual detection tasks while walking for two hours both unloaded and with a 40 kg rucksack load. They found greater errors and longer reaction times during loaded vs unloaded marching, but only after the first hour and with the inclusion of surface grades other than level. Knapick and colleagues^61^ found reduced cognitive performance after a 21 km road march while carrying a heavy rucksack load. Our results suggest that for walking durations of ≤1 hour, and with ≤40% body weight load, there is likely not a negative effect on simple visual oddball task performance with walking.

### Effect of physical demand on cognitive processing

#### Channel level ERPs

At the channel-level of analysis, the largest amplitude and broadest scalp distribution differences were between seated and walking, but not between unloaded and loaded walking conditions. Smaller and more localized differences, at electrodes over the left sensorimotor regions, were found between unloaded and loaded walking conditions. Early aspects of the P300 waveform were similar between all three conditions, but later (>300 ms) event-related potential aspects were affected by the motor task (sitting vs. walking). The effects of dual-task paradigms on performance and P300 waves have been studied for decades^20,21,62,63^. However, walking has only been used in these types of paradigms in the past decade or so^4,8,64,65^ due to the limitations of collecting EEG during movement, particularly walking. The dual-tasking literature suggests that when the P300 generating task is the secondary task, which takes lower priority below some other primary task, the P300 amplitude is reduced relative to performing the task alone. Even further, as the primary task difficulty is increased, often P300 amplitude decreases even further^10,20,21,65^. This is believed to occur because the tasks share resources, and thus when both tasks are performed simultaneously, resources are split between the tasks, and thus more resources are allocated to the primary task. While we did not instruct our participants to prioritize the walking task, our data suggests that they selected this naturally to prevent tripping and falling.

#### Cortical source analysis

Previous research has independently investigated the electrocortical sources involved in walking and P300 eliciting tasks. Studies investigating the neural sources involved in walking have indicated a broad network of cortical sources including parietal, cingulate, sensorimotor, and frontal areas^6,50^. Similarly, studies investigating neural sources involved in P300 tasks have indicated frontal, motor, parietal, cingulate, and temporal regions^4,20,22^. If walking and P300 tasks share cognitive resources as suggested by their electrocortical correlates, it is not surprising that performing the tasks simultaneously would result in changes in the P300 event-related potential.

We found that the left and right parietal regions, sensorimotor regions, and cingulate all demonstrated changes in P300 components amplitude in sitting compared to walking (Fig. 6). We found no significant change in activity in the occipital and frontal regions. This suggests that visual resources are likely not heavily taxed performing a visual oddball task while walking on a treadmill. In the frequency domain, we found differences in all 12 regions of interest, with the largest differences in the left and right parietal, precentral and postcentral gyri (sensorimotor), and cingulate gyri. These differences were primarily at the alpha and beta band frequencies. Pizzamiglio and colleauges^66^, found similar single-task versus dual-tasking walking differences in scalp areas over the posterior/parietal brain regions. However, they did not perform a source-level analysis to assess brain regions responsible for contributing to the differences seen at the scalp level.

#### Limitations

As with any mobile EEG study, there is a possibility that the presence of artifacts could interfere with our interpretation of the data. To minimize this effect, we had our study participants walk at a speed of 1 m/s^24^. We also performed independent component analysis to separate and remove sources of artifact that cannot be removed with traditional filtering alone. We also had subjects focus on crosshairs at the middle of the display to minimize extraneous head movements and the related muscle and movement artifact. Additionally, our analysis focused on the cognitive events, which presumably occur at different times throughout the gait cycles (and thus gait related artifact), such that when we perform ensemble averaging, these artifacts are even further reduced.

Another limitation is that we did not include any conditions where we did not have a button press to indicate identified targets. We cannot tease apart whether the differences between walking only and walking while performing the cognitive taske are due to competing cognitive or motor resources. However, we did begin to see significant differences in neural responses before the button press occurred, suggesting that the responses were the result the cognitive task. In addition, we did not see lateralized differences, which you may expect to see if it were motor interference as subjects were always pressing button with their right hand. Future studies should include cognitive tasks that do not require a motor response.

The lack of effect of time on the walking task should be carefully interpreted. In the scheme of real-world tasks, our paradigm was relatively short, and at 0% grade was moderate intensity. Other studies have found behavioral and neural effects with higher intensity and longer duration manipulations^12,17,48^.

## Conclusions

In our study, we found that subjects used overlapping neural resources for cognitive and motor tasks, which resulted in differences in cortical activation patterns between seated and walking conditions. Importantly, these cortical differences were not correlated with changes in cognitive performance, suggesting that the brain successfully reallocated resources to perform both tasks simultaneously. Even though the subjects’ perceived exertion greatly increased by adding on a 40% body weight load to walking, the cortical dynamics were not fundamentally different in processing the visual oddball task. This suggests that changes in physical load carriage may be modulated at subcortical levels. In future studies, it will be important to explore a combination of tasks that results in a decline in task performance to determine if these neural signatures could be used as a biomarker of task performance and to determine which brain sources are responsible for the decline in task performance.

## Data Availability

The data analyzed during the current study are available from the corresponding author on reasonable request.
